# A new biological and clinical resource for research into pregnancy complications: The Baby Bio Bank

**DOI:** 10.1016/j.placenta.2016.08.085

**Published:** 2016-10

**Authors:** Lydia J. Leon, Nita Solanky, Susanne E. Stalman, Charalambos Demetriou, Sayeda Abu-Amero, Philip Stanier, Lesley Regan, Gudrun E. Moore

**Affiliations:** a UCL Institute of Child Health, 30 Guilford Street, London WC1N 1EH, United Kingdom; b Academic Medical Center, Meibergdreef 9, 1105 AZ Amsterdam, The Netherlands; c Obstetrics and Gynaecology Department, St Mary's Hospital, Imperial College, Praed Street, London W2 1NY, United Kingdom

**Keywords:** Biobank, Pregnancy complications, Preterm birth, Fetal growth restriction, Miscarriage, Pre-eclampsia

## Abstract

About 20% of pregnancies are affected by some form of complication. Research has shown that anomalies in implantation, development, and growth of the fetus; ineffective nutrient exchange between mother and fetus due to placental dysfunction; and maternal problems such as hypertension or infection during pregnancy can all lead to adverse pregnancy outcomes. However, the molecular aetiology of such events remains poorly understood. Fetal growth restriction (FGR), recurrent miscarriage (RM), preterm birth (PTB), and pre-eclampsia (PE) are the most common pregnancy complications encountered in the UK and these outcomes can result in an array of morbidities in both mother and baby, and in the most severe cases in mortality. We need to know more about normal pregnancy and where the important triggers are for failure. This prompted us to collect a large set of biological samples with matching clinical data from over 2500 normal and abnormal pregnancies, for use in research into these conditions. This paper outlines the nature of these sample sets and their availability to academia and industry, with the intention that their widespread use in research will make significant contributions to the improvement of maternal and fetal health worldwide (http://www.ucl.ac.uk/tapb/sample-and-data-collections-at-ucl/biobanks-ucl/baby-biobank).

## Highlights

• The Baby Bio Bank is a new resource for research into complications in pregnancy.
• Over 50,000 samples and linked clinical information for over 2500 pregnancies.
• Dedicated to the improvement of maternal and fetal health nationally and worldwide.
• The resource is open for applications from academic and industrial institutions.

## 1 Introduction

Each year, tens of thousands of women in the UK, and millions across the world, encounter mild to life-threatening pregnancy complications. Despite substantial developments in the clinical management and biological understanding of such cases, many questions regarding the molecular aetiology of common complications in pregnancy remain unanswered. A key challenge often associated with research into pregnancy is obtaining sufficient numbers of biological samples to conduct well-powered studies. The Baby Bio Bank (BBB) is a large, UK based biobank that was set up to remove the obstacle of recruitment and to support research into the environmental and genetic mechanisms underlying common complications of pregnancy. The BBB provides ethically approved, high-quality biological samples and clinical data to academic and industrial partners, both nationally and internationally. Funded primarily through the UK based charity, *Wellbeing of Women*, the BBB began recruiting pregnant mothers from three hospitals across London in 2009. The project's recruitment phase has now reached completion, amassing over 54,000 biological samples from 2515 healthy and complicated pregnancies.

Recruitment and sample collection was based at three London hospitals: Queen Charlotte and Chelsea (QC), Chelsea and Westminster (CW), and St Mary's (SM). Together these London maternity services deliver around 13,000 infants a year. The BBB received ethical approval for collection from these hospitals from Trent Derby Research Ethics Committee [1]. Ethical consent for biological sample collection from the proband (fetus/infant), mother, and father, as well as access to participating patient records was obtained in advance of the birth. All samples are stored at the UCL Institute of Child Health, with a duplicate of the entire biobank located at SM, Imperial College London. Semi-anonymised patient data, downloaded directly from electronic clinical records and supplemented by data gathered by the BBB recruitment team (see S6 for questionnaire format used), is stored in a secure, custom designed online database that links directly to matching sample information.

Fetal growth restriction (FGR), pre-eclampsia (PE), preterm birth (PTB) and recurrent miscarriage (RM) were chosen as the primary foci for the BBB on account of their prevalence within the UK, but also because they capture a physiological spectrum of pregnancy disorders. These disorders are all considered multifactorial, caused by the combined and varying effects of the maternal and fetal genotype, the intrauterine environment, and many other clinical and environmental factors. It is thought likely that many issues covering abnormal implantation to preterm membrane rupture and delivery may share similar or overlapping aetiologies, including physiological mechanisms, pathways and even specific genetic predisposition variants.

The BBB contains data and paired biological samples from 236 FGR, 133 PE, 373 PTB, and 232 RM pregnancies. Over 1500 ‘normal’ pregnancies with none of the above complications were also collected for use as control samples, and 636 of these are classified as ‘perfect’ controls with no recorded problems associated with the mothers' health, pregnancy, or delivery.

A unique and valuable aspect of this dataset is the comprehensiveness of the paternal samples and clinical data that has been amassed, accompanying 68% of the participating pregnancies. Availability of these samples enables investigators interested in genetic contributions to common pregnancy complications to conduct powerful genetic studies using traditional trio designs, or to study in isolation the paternal contribution to pregnancy and fetal outcome.

This paper summarises the design, recruitment, and main demographic and clinical characteristics of the BBB cohort, and provides researchers with contact details to enquire about availability for sample groups of interest. Detailed information regarding sample collection, preparation, and storage are available in Supplementary Information.

## 2 Recruitment design

On account of the expertise of the BBB's founders and the likely high contribution of genetic factors to the phenotypes of interest, the BBB was principally conceptualised as a genetic and epigenetic resource. However, additional opportunities for biomarker research are available due to the parallel collection of plasma/sera from mothers, fathers and infants, in addition to urine from the mother.

**Fig. 1:**
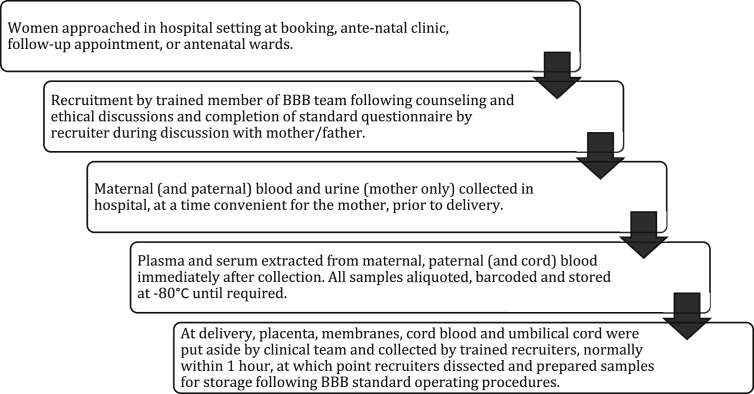
Flow-diagram outlining BBB recruitment and sample collection process.

Recruitment for the BBB was carried out over a four-year period at three hospitals (SM, CW, QC), following a targeted prospective cohort design (Fig. 1). Where possible, recruitment occurred at the antenatal clinic or at follow-up appointments when conditions of interest first presented. This normally gave participants at least several days to ask questions about the consent form and information leaflets. Some women were also recruited on antenatal and labour wards. Any consenting family having a baby at one of the participating units was able to contribute.

Given the ambitious size of the project, a variety of strategies were employed to ensure that recruitment to each complication was as high as possible, whilst still ensuring that they were recruited prior to delivery. Many women were targeted for recruitment if deemed to be at risk for a particular complication. For example, women belonging to certain risk groups (e.g. with a hypertensive disorder, or complicated pregnancy history) were approached by recruiters. Nurses and clinicians would also notify recruiters when women began presenting with clinical symptoms of a complication, regardless of prior risk status (e.g. at the onset of pre-eclampsia or following preterm membrane rupture). In addition, a large number of normal, uncomplicated pregnancies were also recruited for comparative purposes, with some of these pregnancies ultimately contributing to the complications groups (e.g. a woman with no known complication at the time of recruitment could end up delivering preterm).

This process was designed to be feasible within a routine clinical setting. Whereas a simple randomised or stratified randomised design would have been theoretically preferable in minimising selection bias, this was not possible given the context and the resources at hand.

The BBB recruitment team consisted of three full-time recruiters: two research associates with international experience in recruitment and research, and one research nurse with substantial experience of recruitment to large cohort collections, all of whom were trained phlebotomists and had expertise in the necessary sample preparation techniques. Each recruiter was based at one of the three hospitals taking part in the project. The recruiters were responsible for counselling and obtaining consent from the parents, as well as collecting and preparing samples around the clock. Recruitment numbers for each hospital are listed in Table 1. Recruitment was initiated at SM, before recruitment commenced at CW, followed by QC.

## 3 Sample collection and storage

The variety of samples (and relevant accompanying information) that were intended for collection from each pregnancy are presented in Table 2. Recruiters focused on securing the collection of ‘Trio’ sample sets, in which tissue, DNA, and RNA are available for mother, father, and baby. This aim was achieved for 1328 pregnancies in total, across all of the complications and control pregnancies. If we assume a dominant model of inheritance for a potential genetic trait of interest, and a significance threshold of 5%, these trio numbers within the BBB would have reasonable power (>0.7) to detect risk variants with relative risks above 1.5, if all cases are combined into one larger ‘complications group’ (under the hypothesis that certain variants may affect all complications), or 2–2.5 if case groups are considered individually (see S5 for further details).

Each recruitment centre took responsibility for sample receipt and storage. Samples received were matched with the clinical phenotype data and barcoded at point of entry. All tissue and blood samples are stored at −80 °C until requested, with tissue stored in *RNAlater*. DNA and RNA are stored at −80 °C and are available for all samples in which the relevant primary tissue is available.

Each placenta was collected at birth and processed as quickly as possible (normally within one hour). Four 1 cm^3^ sections were excised from beneath the placental membrane, equidistant from the umbilical cord and washed in PBS to remove excess maternal blood. Collecting multiple samples from each placenta increased the total amount of tissue available, as well as facilitating investigations into variation in gene/protein expression in different parts of placenta. By taking samples from four distinct sites, mosaicism can be detected by standard karyotyping. Placental mosaicism is present in approximately 2% of chorionic villous samples [2] and it is likely that a number of such samples will be present in our collection. In addition to sampling from the chorionic plate, villous tissue from the maternal side of the placenta, as well as umbilical cord, cord blood, and fetal membrane tissue were also collected where possible. All tissue was immediately transferred to a vile containing *RNAlater* to minimise degradation of nucleic acids. Occasionally the placenta, cord or cord blood were not available (e.g. a baby was delivered at home, in an emergency, or another hospital). In such instances a buccal swab from the baby was requested and DNA extracted from this specimen. Buccal swabs for DNA extraction were also taken from fathers where blood was unavailable.

For ease of collection and to encourage participation by minimising the number of blood draws/hospital visits for mothers, maternal blood and urine samples were collected once during pregnancy, at a time that was convenient to the mother, and were not restricted to a certain time point in gestation. This usually coincided with hospital appointments and blood draws being taken for clinical purposes. Plasma and serum were extracted immediately from such samples (see S2 for detailed protocol). Where possible, approximately 20 ml of blood was collected from each of the parents. Whole blood, serum and plasma were aliquoted into maximum volumes of 2 ml to minimise exposure of samples to freeze-thaw cycles. Urine was collected in standard specimen containers by the donor and aliquoted into 2 ml volumes within 1 h, for long-term storage at −80 °C. Given the expected high yields of DNA from the protocols used for extraction, and the volume of samples collected, tens of thousands of standard molecular analyses will be possible using the amount of DNA and RNA available within the bank.

**Fig. 2:**
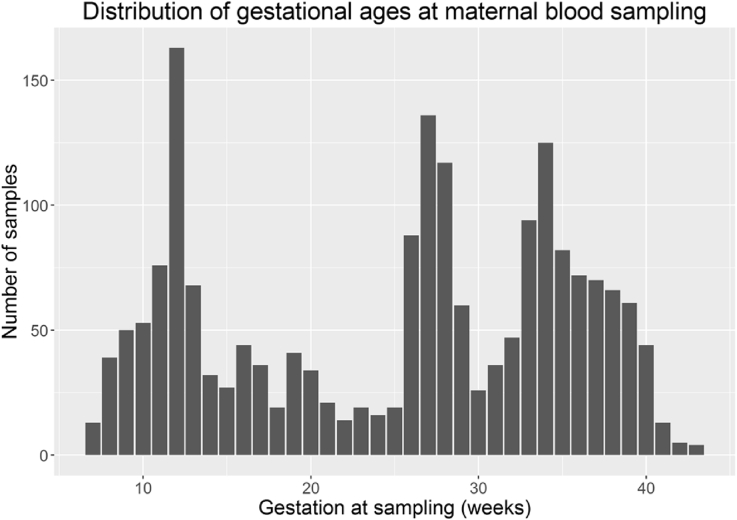
Graph showing distribution of gestational ages at maternal blood sampling within the BBB.

Maternal blood collection ranged from 7 weeks gestation until delivery, with the mean gestational age for collection being 25 weeks. Bloods are available for points collected throughout pregnancy, with peaks at certain times, such as week 12 which coincides with a key antenatal hospital visit for mothers in the UK (Fig. 2). 20% of blood samples were collected within the 1st trimester, 38% in the 2nd, and 42% in the 3rd. BBB sample requests can be specified by gestational age. As maternal samples were collected once from each pregnancy, consecutive samples are not available. We recognise that this, and the variation in time points of blood collection, would be a limitation for certain biomarker studies, however this maximised the number of participants within the study.

To ensure biological samples were processed and maintained to the highest possible standards necessary for use across all common molecular applications, detailed quality control audits using PCR, sequencing, and nucleic acid integrity assessment with the Agilent 2200 TapeStation System, have been conducted on a random selection of at least 15% of the whole cohort. These tests have assured high purity and integrity of DNA and RNA extracted from BBB samples, supporting their use in standard molecular biology assays including Sanger sequencing, qPCR and end-point PCR in which all samples tested positive for housekeeping genes used as standards. A number of samples have also been used successfully for exome analyses and genome-wide methylation assays, in which high DNA quality and integrity is essential.

Both informal and formal audits of the BBB were conducted. Informal audits were conducted by the BBB manager. Formal audits were carried out annually by both the UCL and Imperial Human Tissue Authority committees respectively. On all audits there was 100% concordance with samples in the electronic database and the physical location of samples. This was seen in both directions (from database to sample and sample to database). Retrieval of consent forms for all samples was also 100%.

## 4 Clinical and demographic characteristics

Pregnancy related morbidity and mortality are known to be associated with various neonatal and maternal characteristics that are well documented in the BBB, and may be of interest either as the central phenotype under investigation or in downstream multivariable analyses. To ensure the BBB clinical database was as comprehensive as possible, data were collected by recruiters using standardised forms in addition to detailed clinical downloads from electronic maternity records at each hospital. The two collections were subsequently merged by the BBB data manager into a format that is available to BBB users. This design enabled the collection of certain additional maternal data (e.g. occupation) as well as paternal data, which were not available on clinical records. This strategy also allowed for discrepancies in data entry to be identified and corrected via comparison of any duplicate categories. A selection of the key recorded information and their provenances are listed in Table 3. A full list of clinical data categories is available on request.

The original project aimed to collect only pregnancies from white European mothers and as can be seen (Table 4) this remains the largest group within the BBB. However, due to the multinational population in London it proved difficult to identify and consent a strictly white European cohort. Many clinics were attended by individuals of Asian and African origin expressing an interest in participating in the BBB. We were aware that such a valuable resource would be of greater value if it were inclusive of all ethnic backgrounds and ultimately included such individuals in the hope that funding would one day be available to cover a similarly sized cohort collection for these ethnic groups too.

Ethnicity can be grouped in numerous ways and there is often a lack of consensus within clinical and research communities on precisely how to do this. The BBB electronic database contains two separate data columns on maternal ethnicity, that are used widely throughout the NHS and are based on those used in the national census. Ethnicity data is summarized into four broad categories to give the reader an idea of the ethnic distribution within the BBB cohort. The main demographic characteristics of currently available clinical data of the cohort are outlined in Table 4. Some categories, e.g. delivery method, have larger amounts of missing data than others. The further population of these missing fields is ongoing, requiring the interrogation of paper rather than electronic notes.

## 5 Definitions and verification of outcome data.

Clinical diagnosis of FGR, PE, RM and PTB is recorded for all our samples, the definitions for each of these complications were consistent across the three NHS hospitals from which our samples were collected and are outlined below. However, we recognise that researchers may have specific definitions to categorise clinical complications. To address this, each BBB sample is linked to, and can be searched by more than 200 fields of data that we hold, enabling bespoke categorisation of sample sets, as well as a deeper analysis of data by the creation of sub-categories as required. Whilst this potential for sub-categorisation is a valuable feature of the Bank, we recognise that any such manipulation of the cohort is limited by the original clinical definitions used during recruitment, as stipulated in the following sections.

## 6 Preterm birth

Prematurity affects between 5 and 18% of births worldwide and is the leading cause of neonatal death globally [3]. In the UK, around 7% of births are preterm [4]. Prematurity is associated with a complex array of morbidities from neurodevelopmental issues, to gastrointestinal complications that often extend beyond the neonatal period, through childhood and into adulthood [5]. Preterm births in the BBB were defined as any delivery occurring before 37 weeks gestation.

Biological samples were collected from the 373 preterm pregnancies within the BBB, 122 of which were trios. To achieve this sizeable collection, recruiters actively targeted mothers with a history of preterm birth, women presenting with spontaneous preterm membrane rupture or threatening preterm labour, women with shortened cervices, or those with a positive fetal fibronectin result. Research into the underlying aetiology of preterm birth often focuses on the most extreme ‘very early preterm’ cases in order to best elucidate underlying mechanisms. Generally classified as those born at less than 33 weeks gestation, these births are also the hardest to collect biological material from, given the complicated nature of such deliveries. The BBB has managed to secure biological and clinical data from 96 such valuable cases.

The mean gestational age at birth for this group (Table 5) is 33 weeks and the youngest delivery was at just 20 weeks gestation, 3 weeks below the current ‘limit of viability’ upheld by obstetricians [6]. PTB is often split into three main phenotypic categories for research: spontaneous, indicated and preterm premature rupture of membranes (PPROM). The numbers of pregnancies within each of these sub-categories are also displayed in Table 5. This sub-categorisation was carried out with reference to data from clinical records that provided labour, membrane rupture and delivery information. Definitions used for categorisation are listed below Table 5. The BBB preterm deliveries were fairly evenly distributed between the three main modes of delivery: emergency caesarean section (CS), elective CS, and vaginal.

## 7 Pre-eclampsia

PE is a multisystem disorder that affects around 2–8% of pregnancies globally [7], [8], [9]. Patients typically present with hypertension during pregnancy in conjunction with proteinuria. The condition tends to affect women in the latter half of pregnancy and is associated with both FGR and PTB. PE is estimated to account for 10–15% of all global maternal mortality, with women in developing countries bearing the vast majority of this burden [10].

In our cohort, pre-eclampsia was diagnosed as new hypertension, i.e. at least two consecutive blood pressure (BP) readings above 140/90 mmHg or an increase in systolic BP of at least 30 mmHg or diastolic BP of at least 15 mmHg above booking, combined with new proteinuria defined as a protein creatinine ratio (PCR) of greater than 50, or 24 h quantitation with a level of greater than 300 mg. These diagnoses were recorded in the clinical notes and available to BBB recruiters and data managers for subsequent categorisation. Women who had personal or family history of PE, displayed one or more of the symptoms of the disorder, or were being actively monitored or treated for it were targeted by the recruitment team.

The BBB recruited 133 women who went on to have a pre-eclamptic pregnancy, from which over 700 biological samples were accrued. 41 of these pregnancies were collected as trios. 45 of these women also delivered preterm, representing an important sub-phenotype of the syndrome. As well as women with clinically diagnosed PE, the BBB also contains a substantial number of women with PE associated symptoms such as pregnancy induced hypertension (PIH) or renal complications (as identified from maternal clinical records). The number of pregnancies associated with these morbidities within the BBB are summarized in Table 6.

## 8 Fetal growth restriction

FGR describes the condition of a fetus unable to reach its growth potential [11], [12], which often results in a small for gestational age (SGA) baby. SGA is defined as a birthweight for gestational age below the 10th centile and is often used as a proxy for FGR during pregnancy. In high-income countries, FGR affects about 5–6% of pregnancies [13], [14], [15], [16]. FGR is associated with perinatal morbidity and mortality. Furthermore, survivors are at risk for adult-onset diseases, such as type 2 diabetes, obesity and cardiovascular disease [17], [18], [19], [20].

Although the original intention was to establish a pure FGR cohort, we have chosen to use a broader definition of growth restriction in this summary paper, and report here the characteristics of SGA infants within the BBB, whose birthweight for age centile (as defined by the 1996 gender specific four-in-one growth charts produced by the Child Growth Foundation, London) fell below the 10th centile. Whilst we are aware of the limitations that this definition holds as a proxy for all FGR pregnancies, with the current debate surrounding definitions of FGR and variations in clinical diagnoses we decided to use this looser definition under the assumption that a large number of these SGA babies would indeed be true cases of FGR. Furthermore, more detailed clinical data from the pregnancy, including from ultra-sound scans that enable the mapping of growth trajectories, can be retrieved if a more conservative and strict definition of FGR is required by BBB users.

The BBB recruited 234 women with babies that fell below the 10th birthweight for gestational age centile. 82 of these deliveries were successfully recruited as trios. Within this group, 112 babies were below the 5th birthweight centile and 50% delivered preterm. The mean birthweight for this SGA cohort was 1880g. The distribution of birthweight for age centiles within the BBB, alongside the mean birthweight for each centile grouping are summarized in Table 7.

Alongside interest in the physiological, genetic and environmental underpinnings of FGR, there is now a growing interest in deliveries at the other end of the spectrum in which neonates are large for gestational age, known as macrosomia. Over 200 babies in the BBB were born above the 95th birthweight for age centile and over 300 were above the 90th centile. This group poses particular risks to mothers during delivery and is an important phenotype to study in attempts to minimise maternal morbidity and mortality related to childbirth.

## 9 Recurrent miscarriage

Miscarriage is the commonest complication of pregnancy and is defined as the spontaneous loss of a fetus before it has reached viability. Hence, the term miscarriage includes all the losses from conception up to the 24th week of gestation. Recurrent miscarriage affects about 1.5% of couples trying to conceive [21]. Studies have shown that the risk of miscarriage increases after each successive pregnancy loss and it can reach 45% after three consecutive losses [22]. Recurrent miscarriage (RM) was defined as three or more consecutive pregnancy losses, data that was collected from the clinical records.

The BBB contains samples and clinical data for 232 women who have a clinical diagnosis of RM, as well as many who have experienced one or two previous miscarriages (Table 8). A large number of these cases were recruited from the internationally recognized centre for mothers' suffering from recurrent miscarriage based at SM and run in part by the BBB principal investigator, LR. 97 of the RM pregnancies belong to a BBB trio. In total over 1500 miscarriages were experienced by women in the cohort, and 770 women had had at least one previous miscarriage prior to their BBB pregnancy. 20 women were receiving treatment with aspirin during their pregnancy.

## 10 Other complications

Alongside the four main complications in pregnancy, the size of the BBB means that a substantial number of other maternal complications were also recorded, as detailed in Table 9.

## 11 Using the BBB

Interested parties are encouraged to contact the BBB management team with any questions or requests they may have relating to the bank. Procurement of samples is subject to review by the BBB Research Management Board (who have so far approved all 14 initial applications), as well as written evidence from the applicant of an ethically approved project for which the samples will be used. Enquiries should be directed to the BBB Manager, Dr Nita Solanky (nita.solanky@ucl.ac.uk). Further information on the BBB including our SOPs, contact details and application form are available at http://www.ucl.ac.uk/tapb/sample-and-data-collections-at-ucl/biobanks-ucl/baby-biobank.

## Conflicts of interest

None.

## Figures and Tables

**Table 1:** Hospital recruitment rates.

Hospital	N (%)
St Mary's	1107 (44)
Chelsea and Westminster	880 (35)
Queens Charlotte's and Chelsea	528 (21)

**Table 2:** Variety of samples collected from participating trios.

Sample type
Maternal whole blood/serum/plasma
Paternal whole blood/serum/plasma
Maternal urine
Maternal DNA and RNA (from blood/buccal swab)
Paternal DNA and RNA (from blood/buccal swab)
Placental parenchyma, villous, and membrane
Cord blood
Umbilical cord tissue
Baby DNA and RNA (from placenta/buccal swab)

**Table 3:** Selection of clinical information available.

Maternal	Paternal	Fetal
Age^a^^,^^b^	Age^a^	Gender^b^
BMI^b^	Height^a^^,^^c^	Gestation^b^
Height b^,^^c^	Weight^a^^,^^c^	Birthweight^b^
Weight b^,^^c^	BMI^c^	Placental Weight^c^
Ethnicity^b^	Ethnicity^a^	Head Circumference^b^
Pre-Pregnancy Weight^a^	Diabetes^a^	Birth Length^b^
Parity^a^^,^^b^	Hypertension^a^	Apgar Score^b^
Diabetes^a^^,^^b^	Smoking^a^	Mode Of Delivery^b^
Hypertension^a^^,^^b^	Medication^a^	Congenital abnormalities^b^
Smoking^a^^,^^b^	Occupation^a^	
Medication^a^^,^^b^		
Occupation^a^^,^^b^		
Pregnancy history^a^^,^^b^		
Age at menarche^a^^,^^b^		
Marital status^a^^,^^b^		
Infectious disease^b^		

a Information volunteered from patient to recruitment staff.

b Information gathered from clinical records.

c Measured by recruiting staff.

**Table 4:** Demographic characteristics of the BBB cohort.

Variable	Category	N (%)	Range (SD)	Mean
Maternal ethnicity	White	1565 (68.2)		
	Black	229 (10.0)		
	Asian	99 (4.3)		
	Other^b^	401 (17.5)		
Maternal smoking	Non-smoker	2074 (87.7)		
	Smoker	100 (4.2)		
	Quit within last 12 months	190 (8.1)		
Maternal age at booking	<20	25 (1.3)	14–55 (5.48)	32.83
	20–25	187 (9.6)		
	26–30	372 (19.1)		
	31–35	741 (38.0)		
	36–40	478 (24.5)		
	41–45	140 (7.2)		
	>46	6 (0.3)		
Maternal BMI at booking	<18.5 (Underweight)	71 (3.0)	14–66 (5.27)	24.87
	18.5–24.9 (Normal weight)	1304 (55.3)		
	25.0–29.9 (Pre-obesity)	630 (26.7)		
	30.0–34.9 (Obesity Class 1)	217 (9.2)		
	35–39.9 (Obesity Class 2)	94 (4.0)		
	Above 40 (Obesity Class 3)	42 (1.8)		
Maternal parity	Nulliparous	1324 (53.5)	0–12 (0.95)	0.67
	Primiparous	797 (32.2)		
	Multiparous	354 (14.3)		
Neonate gender	Male	1182 (52.0)		
	Female	1092 (48.0)		
Number of infants	Singletons	2445 (97.2)		
	Twins	70 (2.7)		
Delivery method	CS	716 (42.2)		
	Vaginal	980 (57.8)		
Birthweight (g)			295–5470 (728.92)	3164
GA at birth (weeks)^a^			20–43 (2.81)	38.32

a Assessed from ultra-sound scanning.

b Chinese, other Asian, other black, other, and all mixed groups.

**Table 5:** Number of preterm pregnancies according to gestational age and mode of delivery.

Preterm birth data (cumulative N)									
Gestational age at birth (weeks)	Total	Spontaneous PTB^a^	Spontaneous onset of labour^b^	PPROM^c^	Indicated PTB	Missing data on labour/membrane	Caesarean section delivery^d^	Vaginal delivery	Induced labour
20	1	1	0	1	0	0	0	0	1
21	1	0	0	1	0	1	0	0	1
22	2	0	0	1	0	2	0	0	1
23	4	0	3	4	0	4	0	3	1
24	6	0	4	5	1	5	0	5	2
25	16	8	6	8	2	6	2	7	2
26	15	11	9	10	4	0	6	8	2
27	30	17	16	15	6	7	9	12	3
28	33	22	19	20	11	0	17	14	3
29	41	24	21	21	17	0	24	15	3
30	61	30	27	25	21	10	31	17	3
31	80	37	33	31	32	11	40	20	3
32	96	44	39	36	40	12	49	25	3
33	132	58	51	47	60	14	73	32	3
34	186	80	70	64	86	20	94	43	6
35	252	102	87	85	124	26	121	68	21
36	373	145	113	123	186	42	172	105	48

a Spontaneous labour and/or membrane rupture and delivery before 37 weeks.

b Labour is spontaneous and delivery occurs before 37 weeks.

c Membrane rupture is spontaneous and resultant delivery occurs before 37 weeks.

d Emergency and elective.

**Table 6:** Number of pregnancies associated with a pre-eclamptic phenotype.

Pre-eclampsia phenotype	BBB N
Pre-eclampsia	133
PIH	57
PIH in previous pregnancy/ies	76
Essential hypertension	98
Cardiac complications	15
Renal complications	16
Thrombosis	8

**Table 7:** Distribution of centiles and mean birthweight across the BBB.

Centile	BBB N	Mean birthweight (g)
<5th	112	1555
5th-<10th	122	2179
10th -< 25th	576	2614
25th-<50th	410	2876
50th-<75th	401	3273
75th-<90th	273	3590
90th-<95th	143	3913
>95th	206	4122
Data not available	272	NA

**Table 8:** Distribution of number of previous miscarriages within BBB.

No. previous miscarriages	BBB N
0	1666
1	399
2	139
3	121
4	55
5	32
6	11
7	7
8	2
9	3
10	1
Data not available	79

**Table 9:** Summary of extra clinical categories of interest.

Maternal clinical category	BBB N
Diabetes	23
Gestational Diabetes	31
Pregnancy from In vitro Fertilization	22
Placenta previa	11
Placental abruption	26
Urinary tract infection	36
Thyroid complications	27
Cardiac complications	29
Epilepsy	48
