# Supplementary material for: A new biological and clinical resource for research into pregnancy complications: The Baby Bio Bank
Source: Placenta. 2016 Oct;46:31–7. doi: 10.1016/j.placenta.2016.08.085 (PMC5062948; doi:10.1016/j.placenta.2016.08.085)
Supplement: Supplementary file 1 [file mmc1.docx]

**A new biological and clinical resource for research into pregnancy complications: the Baby Bio Bank**

Lydia J Leon_a_*, Nita Solanky_a_, Susanne E Stalman_b_, Charalambos Demetriou_a_, Sayeda Abu-Amero_a_, Philip Stanier_a_, Lesley Regan_c_, Gudrun E Moore_a_

_a_ UCL Institute of Child Health, 30 Guilford Street, London WC1N 1EH

_b_ Academic Medical Center, Meibergdreef 9, 1105 AZ, Amsterdam

_c_ Obstetrics and Gynaecology Department, St Mary’s Hospital, Imperial College, Praed Street, London W2 1NY

*Corresponding author: Lydia J Leon

UCL Institute of Child Health

30 Guilford Street

London WC1N 1EH

Tel: +44(0)207 905 2159

Email: lydia.leon.11@ucl.ac.uk

**SUPPLEMENTARY INFORMATION**

**S1. Collection of samples**

**Collection of blood for DNA and RNA**

5 ml of blood was collected from donors into EDTA (purple top) tubes. Bloods were aliquoted into multiple vials to a maximum volume of 2ml each to reduce freeze-thaw cycling across the bank. All sample sets, regardless of volume originally collected, were divided into at least 2 aliquots. Bloods were refrigerated and processed within 48 hours.

**Collection of blood for plasma and serum**

5ml of blood was collected in sodium citrate (light blue top) tubes for plasma preparation and 10ml of blood into clotted tubes (red top) for collection of serum.

**Collection of urine**

Urine was collected in standard specimen containers by the donor and aliquoted within 1 hour into at least two 2.5ml aliquots.

**Collection of placental tissue and fetal membrane for DNA and RNA**

Most placentas were collected and processed within an hour of delivery to minimize degradation of RNA and proteins and minimize the effect of hypoxia on gene expression. Samples collected after this time window were used for DNA extraction only.

1 cm^3^ excisions from four sites from the placental chorionic plate surrounding the umbilical cord were taken and rinsed three times in room temperature 1 x PBS (Invitrogen) to remove as much maternal contamination as possible. For a selection of placentas, one sample of villous tissue was also removed from the maternal side of the placenta, and stored as per the parenchyma tissues. Each 1 cm^3^ sample was then immersed in a labelled 5 ml container with 5 volumes of RNALater®. Samples could then be stored at 4°C for 1-3 days if immediate freezing was not possible.

**Cord blood**

Where possible we collected up to 10 ml of whole cord blood (EDTA tubes). All cord blood samples were mixed venous and arterial. In a small subset of cases (~2%) cord blood serum and plasma was also prepared.

**Collection of baby/paternal buccal swab**

Occasionally the placenta, cord and cord blood (and paternal blood) was not available (e.g. baby delivered at home, in an emergency or another hospital). In such instances a buccal swab from the baby/father was requested. The SK-2 Isohelix Swab (SK-2S;Isohelix, Cell Projects) was rubbed against the inside of the cheek for a minimum of 20 seconds and up to one minute. The swab stick was then placed back into the plastic tube, the stick removed so that only the swab containing the buccal cells is in the plastic tube, permitting easier manipulation for DNA extraction.

**S2. Processing of biological specimens**

**Processing of blood for DNA**

DNA from blood was extracted using the Invitrogen iPrep™ Purification system in conjunction with the iPrep™ Card:gDNA Blood and iPrep™ PureLink™ gDNA Blood Kit. A total volume of 350μl of fresh or frozen blood was used for extraction, with expected yields of approximately 10μg, in a final volume of 200μl Elution Buffer (supplied). Samples were processed according to the manufacturer’s instructions.

**Processing of blood for plasma**

The sodium citrate vacutainers containing the blood samples were spun at 815*g* for 10 minutes at 4°C in a Sorval® Legend RT centrifuge. The plasma (in the upper layer) was transferred to a clean 15 ml tube without disturbing the white cells in the buffy coat. The transferred plasma is spun at 3200*g* for 10 minutes at 4°C and then aliquoted into at least two 1ml labelled cryovials. Aliquots were stored at -80°C.

**Processing of blood for serum**

The appropriate vacutainers containing the blood samples for serum were spun for 10 minutes at 1200g in a Sorval® Legend RT centrifuge and the upper aqueous layer then aliquoted into at least 2 x 2ml labeled cryovials. Aliquots were stored at -80°C.

**Processing of blood for RNA**

RNA was extracted from frozen blood using the Invitrogen iPrep™ Purification system in conjunction with the iPrep™ PureLink™ Total RNA kit. 5ml of RBC (red blood cell) Lysis Buffer (supplied) was added to 1ml of fresh whole blood and incubated on ice for 10 minutes with vortexing every three minutes during the incubation period. The sample was then centrifuged for 10 minutes at 4°C at 400*g* in a Sorval® Legend RT centrifuge. The supernatant was discarded and the white blood cell pellet resuspended in 2ml RBC Lysis Buffer (supplied) and mixed well by vortexing before centrifuging as above. After removal of the supernatant, the pellet was resuspended in 600μl Lysis Buffer (supplied) and the cells homogenized. This was then centrifuged in a benchtop centrifuge at 2600*g* for 5 minutes at room temperature. The resulting lysate was transferred to an iPrep™ sample tube and RNA isolated according to the manufacturer’s instructions. RNA was eluted in a volume of 100μl Elution Buffer (supplied).

**Processing tissue for DNA**

DNA from tissue was extracted using the Invitrogen iPrep™ Purification system in conjunction with the iPrep™ Card:gDNA Tissue and iPrep™ ChargeSwitch® gDNA Tissue Kit. Up to 10mg of frozen tissue was homogenized with 1ml Lysis Buffer (supplied). 10μl of *RNase* A (5 mg/ml) (supplied) was added to the homogenate, vortexed and the samples incubated for 10 minutes at room temperature. 20μl Proteinase K (20 mg/ml) (supplied) was added, the samples mixed by vortexing and incubated for 1-3 hours at 55°C. If the homogenate was not clear the Proteinase K incubation was extended overnight. The treated samples were processed according to the manufacturer’s instructions. Approximately 12μg of DNA in 200μl Elution Buffer (supplied) was recovered from 10 mg of tissue.

**Processing tissue for RNA**

RNA from tissue was extracted using the Invitrogen iPrep™ Purification system in conjunction with the iPrep™ Card:Total RNA and iPrep™ PureLink™ Total RNA kit or the iPrep™ Trizol® Plus RNA kit. 50mg of frozen tissue was transferred to a sterile tube on ice and completely immersed in 1ml Trizol® Reagent (supplied) before homogenizing well and incubating at room temperature for 5 minutes. 0.2ml chloroform was added and the sample mixed by inversion for 15 seconds and then centrifuged at 12000*g* for 15 minutes at 4°C in a benchtop centrifuge. After centrifugation, approximately 500μl of inorganic upper phase was transferred to a clean iPrep™ Sample tube and RNA extracted using the iPrep™ PureLink™ Total RNA kit according to the manufacturer’s instructions. RNA was eluted in a volume of 100μl Elution Buffer (supplied).

**Processing of buccal swab for DNA**

DNA from buccal swabs was extracted using the Invitrogen iPrep™ ChargeSwitch® Buccal Cell Kit in conjunction with the iPrep™ Forensic Card. 1ml ChargeSwitch® Lysis Buffer and 10μl Proteinase K were added together to make the Lysis Mix and this was added to the buccal swab in a sterile 1.5ml microcentrifuge tube. The buccal swab was completely immersed in the Lysis Mix and vortexed for 10-15 seconds to facilitate mixing and then incubated for 20 minutes at 37°C. The sample was then transferred to the iPrep Sample and Elution Tube and DNA extracted using the Invitrogen iPrep™ ChargeSwitch® Buccal Cell Kit according to the manufacturer’s instructions. DNA was eluted in a volume of 75 -150μl Elution Buffer (supplied) with an expected yield around 1 - 3µg.

**S3. Sample storage**

**Long term storage of blood**

Whole blood is stored at -80°C.

**Long term storage of tissues**

Tissues in RNAlater® can be stored indefinitely at -20°C but are stored at -80°C to control storage space.

**Long term storage of plasma and serum**

Both plasma and serum are stored at -80°C.

**Long term storage of urine**

Urine is stored at -80°C.

**Long term storage of DNA**

DNA is stored at -80°C.

**Long Term Storage of RNA**

RNA is stored at -80°C.

**S4. Equipment and Reagents**

**Reagents**

The Invitrogen iPrep™ Purification System is an automated nucleic acid purification system with assorted kits containing inclusive reagents.

iPrep™ Card:gDNA Blood (IS-10012)

iPrep™ Card:gDNA Tissue (IS-10013)

iPrep™ Card:Total RNA (IS—10014)

iPrep™ Forensic Card (IS—10011)

iPrep™ PureLink™ gDNA Blood Kit (IS-10005)

iPrep™ ChargeSwitch® gDNA Tissue Kit (IS-10004)

iPrep™ PureLink™ Total RNA (IS-10006)

iPrep™ Trizol® Plus RNA kit (IS-10007)

iPrep™ ChargeSwitch® Buccal Cell Kit (IS-10003)

SK-2 Isohelix Swab Isohelix, Cell Projects (SK-2S)

RNALater® (Ambion)

**Equipment**

-80°C, -20°C and 4°C facilities for storage

Invitrogen iPrep™ Purification System (IS-10000; Invitrogen)

Nanodrop® ND 1000 (Nanodrop Technologies Inc.)

**S5. Example power calculation**

Power was calculated using the online Harvard Genetic Power Calculator [1] with the ‘TDT for discrete traits’ option, assuming a dominant model of inheritance (relative risk of variant is same for heterozygotes as homozygotes). Disease prevalence was kept at 5% to represent the average prevalence for the main pregnancy complications under study, frequency and relative risks of variants were changed, as well as sample size, and power was then calculated.

Under a variety of prevalence and relative risk scenarios (type 1 error 0.05), either in which trios from one complication (N=100) or from all complications (N=300) (under the hypothesis that certain variants may affect all complications), the numbers in the bank would have reasonable power to detect the effects of risk variants with relative risks above 1.5 for the larger cohort and 2-2.5 for the smaller (Table 1).”

**Table 1:** Example of power calculation using BBB samples

| Relative Risk | N=100 | | | N=350 | | |
| --- | --- | --- | --- | --- | --- | --- |
|  | p=0.05 | p=0.1 | p=0.2 | p=0.05 | p=0.1 | p=0.2 |
| 1.25 | 0.14 | 0.16 | 0.19 | 0.1 | 0.31 | 0.38 |
| 1.5 | 0.23 | 0.31 | 0.36 | 0.52 | 0.69 | 0.78 |
| 2 | 0.48 | 0.63 | 0.7 | 0.91 | 0.98 | 0.99 |
| 2.5 | 0.7 | 0.84 | 0.88 | 0.99 | 0.99 | 0.99 |
|  | p=prevalence of risk allele | | | prevalence of phenotype=5% | | |

**S6. Standard BBB questionnaire**

**Figure S1:** Example of questionnaire used by BBB recruiters for additional information collected per trio

**Bibliography**

[1] Purcell S, Cherny SS and Sham PC. Genetic Power Calculator: design of linkage and association genetic mapping studies of complex traits. Bioinformatics. 2003;19(1):149-50.
